# Supplementary material for: Zika virus infection in early pregnancy increases the likelihood of persistent neutralizing antibodies
Source: PLoS Negl Trop Dis. 2026 Jan 8;20(1):e0013906. doi: 10.1371/journal.pntd.0013906 (PMC12810899; doi:10.1371/journal.pntd.0013906)
Supplement: Attachment — Submitted filename: ResponseLetter_R1.pdf [file pntd.0013906.s002.pdf]

Submission ID **PNTD-D-25-01416**

Manuscript: "**Zika Virus Infection in Early Pregnancy Increases the Likelihood of Persistent Neutralizing Antibodies**"

Dear Editors Md. Kamrujjaman, (Academic Editor), Michael Holbrook (Section Editor), Shaden Kamhawi (co-Editor-in-Chief), and Paul Brindley (co-Editor-in-Chief),

We would like to thank PLOS Neglected Tropical Diseases and the reviewers for their thoughtful evaluation of our manuscript and for the opportunity to once more revise and improve our work.

All suggested corrections have been incorporated into the manuscript. As requested, the response letter indicates the line numbers where the changes were made, and a file with tracked changes has been submitted as a supplementary document.

Please, note that our following responses are in blue.

==

Reviewers' comments:

Reviewer's Responses to Questions

Key Review Criteria Required for Acceptance?

As you describe the new analyses required for acceptance, please consider the following:

Methods

-Are the objectives of the study clearly articulated with a clear testable hypothesis stated?

-Is the study design appropriate to address the stated objectives?

-Is the population clearly described and appropriate for the hypothesis being tested?

-Is the sample size sufficient to ensure adequate power to address the hypothesis being tested?

-Were correct statistical analysis used to support conclusions?

-Are there concerns about ethical or regulatory requirements being met?

Reviewer #1: Everton Falcão de Oliveira and colleagues evaluated the persistence of ZIKV neutralizing antibodies during pregnancy, which were evaluated 3-4 years after

infection. This study included evaluations of children and mothers. The methodology consisted of collecting epidemiological data and data on the gestation period and birth of the children. The evaluation of ZIKV infection during gestation was carried out by the same team and included a series of assessments to determine the incidence of infection. The evaluation of nABs was performed at a single point in time between 3 and 4 years after birth. The population is well described and is appropriate for this type of study. The sample size is a limitation, but it is understandable for such epidemiological studies, particularly given the sample collection and the epidemiological information available. The statistical analysis was appropriate. and there are no concerns about compliance with ethical requirements.

Author response: We thank the reviewer for this insightful and constructive comment, which helped us to improve our manuscript. We carefully considered all the points raised, as detailed below.

Introduction:

Line 89: What do you mean when you say that new birth cohorts are reaching reproductive age and how this may affect future epidemics? I believe this happens every year before, during, and after the pandemic, and I don't think it's relevant to mention it.

Author response: We thank the reviewer for this observation. Indeed, the original sentence did not convey the intended meaning clearly. We have revised it to clarify our point, which was that individuals who were children or adolescents during the 2015–2016 ZIKV epidemic are now reaching reproductive age without prior exposure to the virus. This renewal of the susceptible population may contribute to the risk of future outbreaks. The revised text now reads as follows (**page 4, lines 87–91, manuscript in tracking format**):

"*Although the incidence of CZS declined substantially after 2017, the threat of future ZIKV epidemics remains, as many populations are still immunologically naive. Moreover, individuals who were children or adolescents during the 2015–2016 epidemic are now reaching reproductive age without prior exposure to the virus, thereby expanding the pool of susceptible individuals and potentially facilitating new outbreaks*".

Regarding the collection of epidemiological data on pregnancy and birth. Was any type of quality control carried out to guarantee the information? Who was responsible for

receiving or storing it? To ensure the processing of the information, it would be important to provide these details.

Author response: We have now added details clarifying the sources and quality control procedures for the epidemiological and obstetric data. These data were extracted from maternal and child health booklets, which are official records completed by healthcare professionals during prenatal and child care visits, and from the Brazilian Live Birth Information System (SINASC). Information on birth and maternal outcomes was cross-checked between both sources to ensure accuracy, and any discrepancies were resolved by prioritizing the data from the maternal health booklet, which is considered the primary record. The revised text now reads as follows (**page 9, lines 216-223, manuscript in tracking format**):

Data were obtained from maternal and child health records, including the prenatal and child health booklets (official documents completed by healthcare professionals during prenatal consultations and child follow-up visits) as well as from the Brazilian Live Birth Information System (SINASC). Information on birth outcomes and maternal complications was available in both sources, and our research team systematically cross-checked these data to ensure consistency. In cases of discrepancies, the information recorded in the maternal health booklet was prioritized, as it represents the primary source completed at the point of care.

Reviewer #2: The authors evaluated the persistence of neutralizing antibodies in antenatal ZIKV infections 3-4 years after infection. Their most important findings reveal that maternal antibody titers persist and are higher when infection occurred in the first trimester; data from infants are inconclusive. The study design and statistical analyses is according to the objective state.

Author response: We thank the reviewer for this insightful and constructive comment.

Reviewer #3: First, I appreciate the opportunity to review an interesting and robust study. My considerations will be brief and to the point, to contribute to the structural clarity of the text.

Author response: We thank the reviewer for this insightful and constructive comment, which helped us to improve our manuscript. We carefully considered all the points raised, as detailed below.

Method: In the "study design" section, keep only the description of the structure and type of research that was carried out and the method applied (it is cited in the abstract as a cross-sectional serological study). I suggest moving the paragraph on lines 118-128 to the introduction, as well as the hypothesis, which should be implied in the section prior to the method.

Author response: As requested by the reviewer, we have moved this section of the Methods to the Introduction, which is now in **lines 125 to 135, page 5-6**, of the manuscript in tracking format and have mentioned the cross-sectional serological study design in the methods (page 6, line 142). The hypothesis section in the Introduction now reads:

"We hypothesize that neutralizing antibodies against ZIKV in women tend to wane over time following infection, as observed in previous studies [9], but that this decline may occur slowly, with a baseline level of antibodies being maintained, as observed for other mosquito-borne flaviviruses, such as certain dengue virus (DENV) serotypes [15,16]. This sustained low-level immunity may explain the absence of Zika epidemics in Brazil since 2017 [6,7]. Potentially, the timing of infection during pregnancy may influence the durability of the neutralizing antibody response, as this factor has been frequently reported as a predictor of the occurrence of congenital Zika syndrome. For children, given the low frequency of anti-ZIKV antibody previously positivity reported [10], we hypothesize that long-lasting immunity against the virus does not occur, particularly when exposure occurred in utero."

Cite the reference for the statement: "This sustained low-level immunity could help explain the absence of Zika epidemics in Brazil since 2017" and include it in the introduction as well.

Author response: We have added two reference for this statement and included it in the introduction.

I suggest, if the authors agree, dividing the objective, since throughout the text association and persistence are evaluated separately. Dividing the objective into persistence of maternal nAbs and the association presented in the hypothesis.

Author response: We thank the reviewer for this observation. We agree that presenting the study objectives separately improves clarity. We have therefore revised the sentence to explicitly indicate the two components of the study aim. The revised text now reads as follows (**page 5, lines 122-125, manuscript in tracking version**):

"*The aims of this study were twofold: (1) to assess the persistence of ZIKV neutralizing antibodies (nAbs) in women infected during pregnancy and in their children with antenatal ZIKV exposure; and (2) to explore whether maternal ZIKV nAb titers were associated with child health outcomes*."

Results:

-Does the analysis presented match the analysis plan?

-Are the results clearly and completely presented?

-Are the figures (Tables, Images) of sufficient quality for clarity?

Reviewer #1: Results. The analyses are consistent with the plan presented.

Table 1. The N of the columns does not match the N of each estimated variable.

Although few children had ZIKV antibody titers, information on breastfeeding could be included, as it may influence the results. The main limitations have already been described, primarily that the analysis is performed at a single point in time.

Author response: We thank the reviewer for this observation. The differences in the total number of observations (N) across variables in Table 1 are due to missing data for a few participants. Specifically, two pregnancies resulted in miscarriage, and therefore, data on early and long-term child outcomes were not available for these cases.

Additionally, information on the gestational timing of maternal ZIKV infection was not available for three women who were asymptomatic during pregnancy. We have added a footnote to Table 1 and comments in the description of the results, as following:

"*Asymptomatic infections (n = 3) were not included in this part of the analysis*" (**page 13, lines 291-292, tracking version of manuscript**).

"*Clinical evaluations for the diagnosis of these outcomes were performed in 75 children, as two pregnancies ended in miscarriage*" (**page 13, lines 300-301, tracking version of manuscript**).

Regarding breastfeeding, this information was not systematically collected in the original study protocol and, therefore, could not be included in the present analysis.

Reviewer #2: The analysis matches the plan designed, the results were presented clearly, and the figures summarize the main results.

Author response: We thank the reviewer for this insightful and constructive comment.

Reviewer #3: The results are described clearly and objectively and meet the objectives set forth at the time of reading. The boxplot is well-suited for use. On line 295: would it be "first quarter" where it says just "quarter"? I believe this also occurred on line 305.
Author response: We thank the reviewer for the careful reading of our manuscript. However, the term "quarter" does not appear in the current version of the text. We have carefully reviewed the manuscript and confirmed that no such term is present.

I would like a justification and explanation for the use of children's ages in months, we usually use weeks or months to account for gestational age and years after birth.
Author response: We used months to describe the ages of children in our cohort so that the methodology used for reporting pediatric age is consistent with that of our prior publications and that of similar Zika pediatric cohorts. Many studies relating to neurodevelopmental outcomes of children with antenatal exposure to Zika virus use months for a child's age rather than years because neurodevelopmental tools have cut-offs for use up to a certain age in months and not years.

Conclusions

-Are the conclusions supported by the data presented?

-Are the limitations of analysis clearly described?

-Do the authors discuss how these data can be helpful to advance our understanding of the topic under study?

-Is public health relevance addressed?

Reviewer #1: The conclusions are well developed in accordance with the objectives set, but the limitations of the study are not mentioned.
Author response: We thank the reviewer for this comment. The limitations of our study were already included in the Discussion section, as follows (**page 19, lines 372-375, tracking version of manuscript**):
"*Our study has limitations, including the lack of evaluation for antibodies against other arboviruses that are endemic in the study region, such as dengue. Additionally, we did*

not have access to acute-phase samples from the mothers during infection, and therefore could not perform longitudinal analyses to assess ZIKV nAb titers over time.”

However, based on your comments, we have added this new statement (**page 19, lines 376-379**):
“The relatively small sample size may also limit the generalizability of our findings. Future studies with larger cohorts and more comprehensive datasets, including information on additional maternal, perinatal, and postnatal factors, such as breastfeeding practices, are needed to strengthen the evidence on the persistence and effects of ZIKV nAbs.”

Reviewer #2: The conclusions are supported by the data presented, and the limitations are clearly specified, such as not presenting an analysis of other arboviruses endemic to the study area and not presenting a longitudinal analysis of the neutralization assays. They demonstrate the importance of conducting an analysis of neutralizing antibody persistence for new antibody-based therapies and counteracting the effects of future epidemics.
Author response: We thank the reviewer for this insightful and constructive comment.

Reviewer #3: The discussion and conclusion meet the objective and proposed hypothesis.
Author response: We thank the reviewer for this insightful and constructive comment.

Editorial and Data Presentation Modifications?
Use this section for editorial suggestions as well as relatively minor modifications of existing data that would enhance clarity. If the only modifications needed are minor and/or editorial, you may wish to recommend “Minor Revision” or “Accept”.

Reviewer #1: My opinion on the editorial and data presentation modifications is “minor revisions,” as it is suggested that data on the breast milk of the children studied be included. Due to the absence of conclusive data on the children's neutralizing antibodies, the conclusions will not change, but the insertion of this data is relevant.
Author response: We thank the reviewer for this comment. Data on the children’s neutralizing antibodies (nAbs) are already presented in the Results section (**pages 16-17,**

lines 313-320, tracking version of manuscript) and in **Figure 1**. As described, PRNT was performed using samples from 10 children, while 62 children had negative results for ZIKV by both IgM/IgG serology and RT-PCR (Figure 1). Among the 10 tested, two had ZIKV nAb titers at levels considered protective. Although the small number of samples limits further subgroup analyses, these findings are included and discussed in the manuscript. Information on breastfeeding, however, was not systematically collected in the study protocol and, therefore, could not be analyzed.

Reviewer #2: (No Response)

Reviewer #3: No further modifications.

Summary and General Comments

Use this section to provide overall comments, discuss strengths/weaknesses of the study, novelty, significance, general execution and scholarship. You may also include additional comments for the author, including concerns about dual publication, research ethics, or publication ethics. If requesting major revision, please articulate the new experiments that are needed.

Reviewer #1: The study of the duration of ZIKV nABs after infection in utero, as well as their relationship to short- and long-term adverse effects in children, is relevant. The authors also argue the importance of such studies for vaccine development and preventing serious effects in future epidemics. Some of the limitations of the study are the sample size and the inclusion of some data that could improve the evidence presented here.

Author response: We thank the reviewer for these thoughtful comments and for recognizing the relevance of our study. We acknowledge that the relatively small sample size and the absence of certain maternal and postnatal variables may limit the strength of the evidence. In response, we have revised the Discussion to better highlight these limitations and to emphasize the need for future studies with larger cohorts and more comprehensive datasets, including information on breastfeeding practices and other perinatal factors.

Reviewer #2: The ZIKV epidemic was a key moment in the search for alternatives to this virus in order to reduce or eradicate its effects on the neurodevelopment of children during

pregnancy. At the time, there was nothing else to do but observe the effects and study the areas that should be explored in depth. Now there is evidence of long-term manifestations, but rather than knowing what happens, it is more important to know what to do to manage future epidemics. This study presents evidence of the persistence of neutralizing antibodies in mothers, which is relevant for therapies that can reduce the effects on children in future epidemics.

Author response: We sincerely thank the reviewer for these encouraging and insightful comments. We fully agree that understanding the persistence of neutralizing antibodies is crucial to guide preventive and therapeutic strategies in future ZIKV epidemics. We appreciate the reviewer's recognition of the relevance of our findings in contributing to this broader public health perspective.

Reviewer #3: I suggest describing the role of neutralizing antibodies in more detail in the Introduction.

I suggest describing the specifics of the immune response in management in the Introduction so that it can be revisited in the discussion. This creates an interesting thread.

Author response: We thank the reviewer for this suggestion. In response, we have expanded the Introduction to provide a clearer description of the role of neutralizing antibodies (nAbs) and their relevance to the immune response to ZIKV infection. This addition helps to better contextualize the study objectives and provides a logical connection to the discussion of our findings. The revised text now reads as follows (**page 5, lines 103-110**):

"Neutralizing antibodies play a central role in protective immunity against ZIKV, preventing viral entry into host cells and contributing to viral clearance [9,10]. However, the magnitude and duration of these responses can vary substantially among individuals. Factors such as the timing of infection during pregnancy, the presence of cross-reactive antibodies to other flaviviruses, and the immaturity of the neonatal immune system may influence the persistence and effectiveness of these antibodies [9,10]. Understanding the dynamics of nAbs is therefore essential to assess long-term immunity, susceptibility to reinfection, and potential implications for vaccine design."
